# New records of German Scelionidae (Hymenoptera: Platygastroidea) from the collection of the State Museum of Natural History Stuttgart

**DOI:** 10.3897/BDJ.9.e69856

**Published:** 2021-09-09

**Authors:** Jessica Awad, Cristina Vasilita, Sophie Wenz, Hamdow Alkarrat, Olaf Zimmermann, Claus Zebitz, Lars Krogmann

**Affiliations:** 1 State Museum of Natural History, Stuttgart, Germany State Museum of Natural History Stuttgart Germany; 2 Alexandru Ioan Cuza University, Iasi, Romania Alexandru Ioan Cuza University Iasi Romania; 3 Institute of Phytomedicine, University of Hohenheim, Stuttgart, Germany Institute of Phytomedicine, University of Hohenheim Stuttgart Germany; 4 Center for Agricultural Technology Augustenberg, Karlsruhe, Germany Center for Agricultural Technology Augustenberg Karlsruhe Germany

**Keywords:** parasitoid wasps, DNA barcoding, *Trissolcus*, *Paratelenomus*, dark taxa

## Abstract

**Background:**

Scelionid wasps are arthropod egg parasitoids, many of which are relevant to global biosecurity. However, the scelionid fauna of Germany has not received much attention from professional taxonomists.

**New information:**

Eleven species and four genera are recorded for the first time from Germany, including species of interest to agriculture and biological control. First genus records include *Baryconus* Förster, *Macroteleia* Westwood, *Paratelenomus* Dodd and *Probaryconus* Kieffer. First species records include *B.europaeus* (Kieffer), *Idrisnigroclavatus* (Kieffer), *Idrissemiflavus* (Kieffer), *M.bicolora* Kieffer, *M.pannonica* Szabo, *Paratelenomussaccharalis* (Dodd), *Trimorusvaricornis* (Walker), *Trissolcusbasalis* (Wollaston), *Trissolcusbelenus* (Walker), *Trissolcuscolemani* (Crawford) and *Trissolcusflavipes* (Thompson). COI barcodes are identified for the first time from *B.europaeus* and *M.bicolora*. Each species is illustrated and updated world distributions are provided. Implications for agriculture are discussed.

## Introduction

Platygastroidea is the third largest superfamily of Hymenoptera in terms of the number of described species, exceeded only by Ichneumonoidea and Chalcidoidea. The current number of valid species is ca. 6,500, with a worldwide estimate of about 10,000 (Hymenoptera Online 2020, Masner 1993, Johnson 2011). At the time of writing, the superfamily comprises two extant families, Scelionidae and Platygastridae (Talamas and Buffington 2015, Popovici et al. 2017).

In the 19th century, several notable experts published on German Platygastroidea. The earliest was Christian Gottfried Nees von Esenbeck, who described dozens of species in *Sparasion*, *Scelio*, *Platygaster* and *Teleas* (Nees von Esenbeck 1834). Julius Theodor Christian Ratzeburg described species of *Platygaster* and *Teleas* associated with forest pests (Ratzeburg 1852). Soon after, Arnold Förster published his "Hymenopterologische Studien", establishing 26 platygastroid genera, many of which remain valid today (Förster 1856). In the early 20th century, Jean-Jacques Kieffer described numerous genera and species from central Europe, including German material (Kieffer 1926).

Since Kieffer, there has not been much research on the Platygastroidea of Germany. The most recent catalogue of German insects (Dathe et al. 2001) lists 136 platygastroid species, including 56 Scelionidae. However, these numbers are certainly low. The section was based on a relatively short reference list and many common European taxa were not included. Thus, Platygastroidea has been identified as a priority for research within the German Barcode of Life III: Dark Taxa project (Hausmann et al. 2020). Dark taxa are insect groups, mainly in Hymenoptera and Diptera, which pose a taxonomic impediment to biodiversity studies. Such taxa are abundant and diverse in insect monitoring projects, but a lack of usable diagnostic literature makes species identification difficult to impossible.

Scelionid wasps parasitise the eggs of arthropods, including many invasive or noxious pest species (Austin et al. 2005). Thus, their accurate identification is critical to agricultural research, especially in the context of the global plant trade. For example, the brown marmorated stink bug, *Halyomorphahalys* Stål, 1855, is an invasive species in Europe and North America. Its most effective natural enemy, *Trissolcusjaponicus* (Ashmead, 1904), has been detected or established as an adventive species throughout the introduced range (Talamas et al. 2015, Abram et al. 2019, Stahl et al. 2019). Most recently, *T.japonicus* was detected in Germany (Dieckhoff et al. 2021). Similarly, *Paratelenomussaccharalis* (Dodd, 1914) has followed the kudzu bug, *Megacoptacribraria* (Fabricius, 1798), from the Palearctic into North America (Gardner et al. 2013).

The current work represents a first update to the German platygastroid fauna within the German Barcode of Life (GBOL) III initiative. As these findings occurred within the first several months of the project, further discoveries are expected over the next three years. Identification of Platygastridae is still underway, as the state of taxonomic disarray in this group is more severe.

## Materials and methods

We examined recent and historical collections of Scelionidae at the State Museum of Natural History Stuttgart (SMNS). Recent material was collected for earlier stages of the GBOL project or for long-term insect monitoring programmes, generally by Malaise trap. Recently-collected specimens were preserved in 96% ethanol. Specimens collected for the GBOL project had DNA extracted non-destructively with the DNeasy Blood & Tissue Extraction Kit from Qiagen following the updated protocol provided by Cruaud et al. (2019). COI barcodes were amplified by PCR with the LCO1490/HCO2198 primers (Folmer et al. 1994). Barcode sequences are available at GenBank accession numbers MW829349–MW829358.

Illustrations were created with a Keyence imaging system. Adobe Photoshop was used for image processing and plate construction.

## Taxon treatments

### Baryconus europaeus

(Kieffer, 1908)

E836DBB2-FC5C-5087-8B97-66E095BE47F5

Hoploteleia europaea Kieffer, 1908
Hoploteleia graeffei Kieffer, 1908
Baryconus graeffei (Kieffer): Kieffer, 1926
Baryconus europaeus (Kieffer): Bin, 1974

#### Materials

- **Type status:** Other material. **Occurrence:** recordedBy: L. Krogmann; individualCount: 3; sex: female; **Taxon:** scientificName: *Baryconuseuropaeus* (Kieffer, 1908); **Location:** country: Germany; stateProvince: Baden-Württemberg; municipality: Markgröningen; locality: Entomological Society of Stuttgart property in the Rotenacker; verbatimLocality: EVS-Vereinsgrundstück am Rotenacker; verbatimElevation: 280 m; **Identification:** identifiedBy: Cristina Vasilita; **Event:** samplingProtocol: sweep net; year: 2009; month: 8; day: 4; **Record Level:** type: PhysicalObject; bibliographicCitation: *Baryconuseuropaeus* (SMNS_Hym_Sce_001093, 1094, 1095); institutionCode: SMNS; basisOfRecord: PreservedSpecimen
- **Type status:** Other material. **Occurrence:** recordedBy: T. Kothe, M. Engelhardt, C. König; individualCount: 1; sex: female; associatedSequences: GenBank: MW829358; **Taxon:** scientificName: *Baryconuseuropaeus* (Kieffer, 1908); **Location:** country: Germany; stateProvince: Baden-Württemberg; municipality: Tübingen; verbatimCoordinates: 48.504317°N, 8.9956°E; **Identification:** identifiedBy: Cristina Vasilita; **Event:** samplingProtocol: Malaise trap; year: 2014; month: 7; day: 17–31; **Record Level:** type: PhysicalObject; bibliographicCitation: Baryconuseuropaeus (SMNS_Hym_Sce_000715); institutionCode: SMNS; basisOfRecord: PreservedSpecimen

#### Distribution

**Figure 1.**
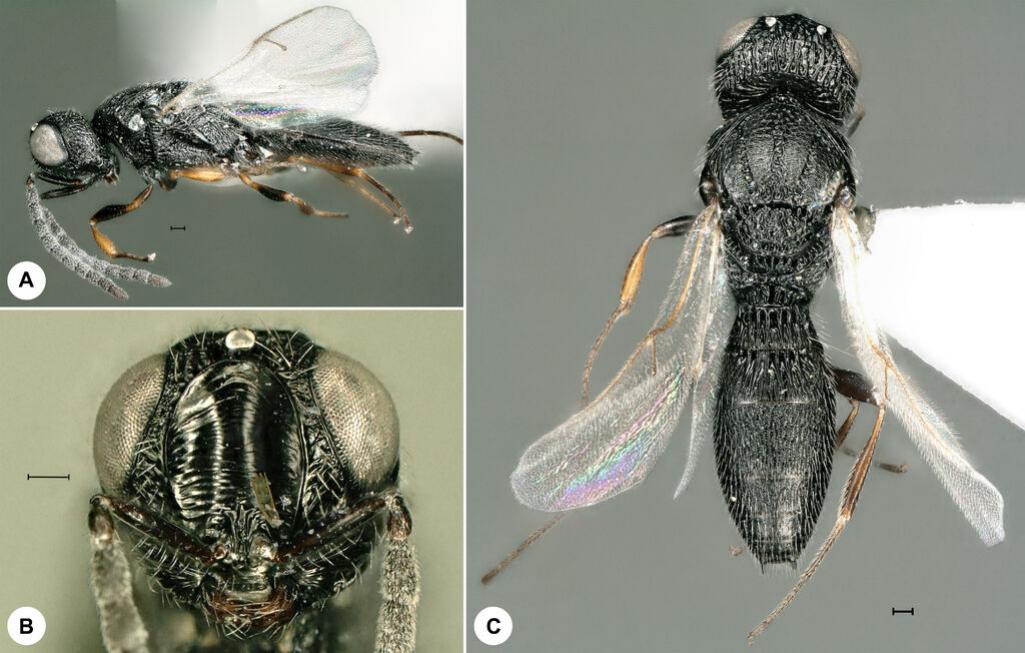
*Baryconuseuropaeus* (Kieffer), female, SMNS_Hym_Sce_1093. **A.** Lateral habitus; **B.** Head, frontal view; **C.** Dorsal habitus. Scale bar = 100 µm.

*Baryconuseuropaeus* (Fig. 1) was described from Italy and has also been recorded from Croatia, Cyprus, France, India, Japan, Morocco, Portugal, Russia, Spain, Turkey and UAE (Popovici et al. 2013). It is expected in Romania (Spiridon et al. 2019). We here provide the first genus and species record for Germany and the first identified barcode for *Baryconuseuropaeus.* Identification is based on Popovici et al. (2013).

### Idris nigroclavatus

(Kieffer, 1908)

CD88CF46-FB53-5F2B-A453-0CB3C358A516

Acolus nigroclavatus Kieffer, 1908
Acolus striativentris Kieffer, 1909
Acolus coxalis Kieffer, 1912
Idris coxalis (Kieffer): Szabo, 1965
Idris striativentris (Kieffer): Kozlov, 1978
Idris nigroclavatus (Kieffer): Huggert, 1979

#### Materials

- **Type status:** Other material. **Occurrence:** recordedBy: J. Reibnitz; individualCount: 2; sex: female; **Taxon:** scientificName: *Idrisnigroclavatus* (Kieffer, 1908); **Location:** country: Germany; stateProvince: Baden-Württemberg; municipality: Markgröningen; locality: Rotenacker Forest east; **Identification:** identifiedBy: Cristina Vasilita; **Event:** samplingProtocol: sieve; year: 2019; month: 4; day: 2; habitat: maple, forest edge; **Record Level:** type: PhysicalObject; bibliographicCitation: *Idrisnigroclavatus* (SMNS_Hym_Sce_001098, 1099); institutionCode: SMNS; basisOfRecord: PreservedSpecimen

#### Distribution

**Figure 2.**
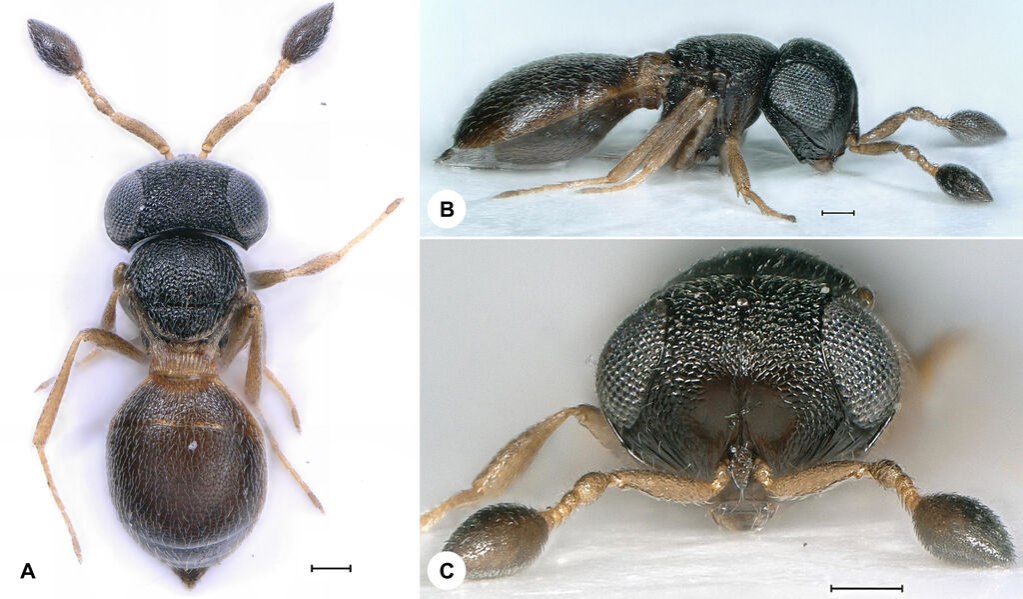
*Idrisnigroclavatus* (Kieffer), female, SMNS_Hym_Sce_001098. **A.** Dorsal habitus; **B.** Lateral habitus; **C.** Head, frontal view. Scale bar = 100 µm.

*Idrisnigroclavatus* (Fig. 2) was described from Italy and has also been recorded from Austria, Bosnia and Herzegovina, Bulgaria, Croatia, France, Hungary, Spain and Sweden (Huggert 1979, Kononova and Kozlov 2001). We here provide the first species record for Germany. Identification is based on Huggert (1979).

### Idris semiflavus

(Kieffer, 1908)

B6C0C640-8847-530D-81AD-61BE4EFFC84B

Acolus semiflavus Kieffer, 1908
Idris semiflavus (Kieffer): Huggert, 1979

#### Materials

- **Type status:** Other material. **Occurrence:** recordedBy: O. Zimmermann, S. Wenz, M. Renninger, A. Reißig; individualCount: 1; sex: female; associatedSequences: Genbank: MZ334547; **Taxon:** scientificName: *Idrissemiflavus* (Kieffer, 1908); **Location:** country: Germany; stateProvince: Baden-Württemberg; municipality: Weil am Rhein; verbatimCoordinates: 47.579614°N, 7.606160°E; **Identification:** identifiedBy: Klaus Schrameyer; **Event:** samplingProtocol: suction sampler; year: 2020; month: 7; day: 14; habitat: ruderal area dominated by *Ailanthusaltissima*; **Record Level:** type: PhysicalObject; bibliographicCitation: *Idrissemiflavus* (SMNS_Hym_Sce_001147); institutionCode: SMNS; basisOfRecord: PreservedSpecimen
- **Type status:** Other material. **Occurrence:** recordedBy: O. Zimmermann, S. Wenz, M. Renninger, A. Reißig; individualCount: 1; sex: female; associatedSequences: Genbank: MZ334548; **Taxon:** scientificName: *Idrissemiflavus* (Kieffer, 1908); **Location:** country: Germany; stateProvince: Baden-Württemberg; municipality: Weil am Rhein; verbatimCoordinates: 47.586876°N, 7.619260°E; **Identification:** identifiedBy: Klaus Schrameyer; **Event:** samplingProtocol: suction sampler; year: 2020; month: 7; day: 14; habitat: ruderal area dominated by *Paulownia* sp.; **Record Level:** type: PhysicalObject; bibliographicCitation: *Idrissemiflavus* (SMNS_Hym_Sce_001148); institutionCode: SMNS; basisOfRecord: PreservedSpecimen
- **Type status:** Other material. **Occurrence:** recordedBy: O. Zimmermann, M. Trautmann; individualCount: 1; sex: female; associatedSequences: Genbank: MZ334549; **Taxon:** scientificName: *Idrissemiflavus* (Kieffer, 1908); **Location:** country: Germany; stateProvince: Baden-Württemberg; municipality: Konstanz; **Identification:** identifiedBy: Klaus Schrameyer; **Event:** samplingEffort: suction sampler; year: 2020; month: 8; day: 7; habitat: ruderal area near apple production; **Record Level:** type: Physical Object; bibliographicCitation: Idrissemiflavus (SMNS_Hym_Sce_001149); institutionCode: SMNS; basisOfRecord: PreservedSpecimen

#### Distribution

**Figure 3.**
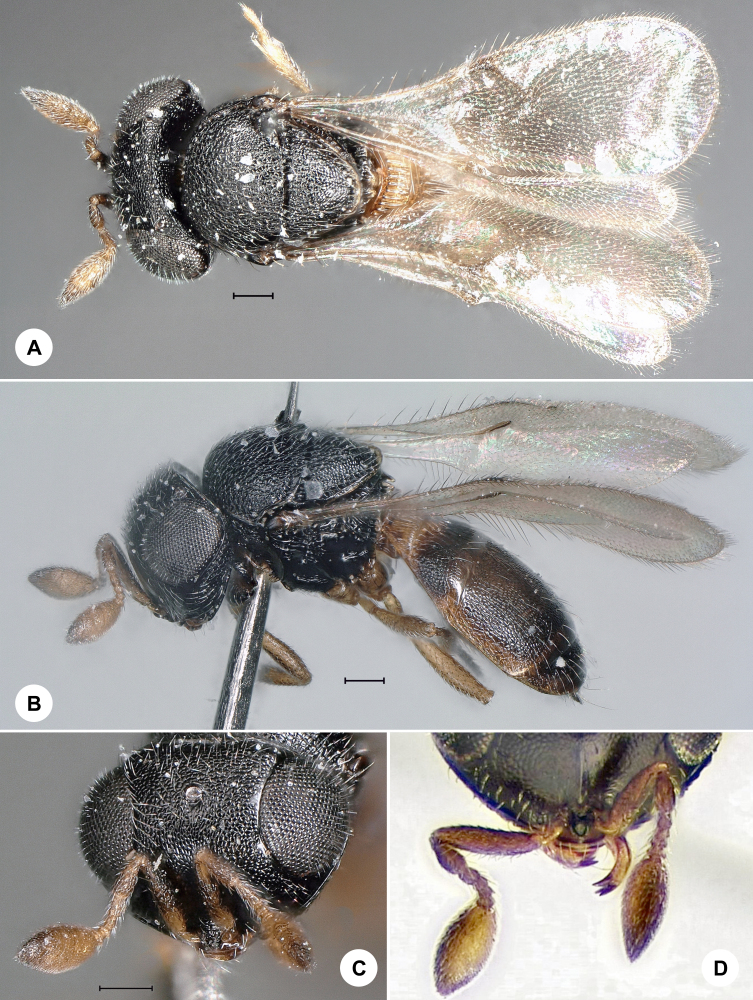
*Idrissemiflavus* (Kieffer), female, SMNS_Hym_Sce_001149. **A.** Dorsal habitus; **B.** Lateral habitus; **C.** Head, frontal view; **D.** Clava and mandible. Scale bar = 100 µm.

*Idrissemiflavus* (Fig. 3) was described from France and has been recorded from Egypt, Hungary, Italy, Mongolia, Spain and Switzerland (Huggert 1979). We here provide the first species record for Germany. Identification is based on Huggert (1979).

### Macroteleia bicolora

Kieffer, 1908

C75C0263-F32F-59CF-95BE-4B93A51F17B0

Macroteleia bicolora Kieffer, 1908
Macroteleia bicolor (Kieffer): Kozlov, 1978

#### Materials

- **Type status:** Other material. **Occurrence:** recordedBy: Patricia Gut; individualCount: 2; sex: female; associatedSequences: GenBank: MW829349, 829350; **Taxon:** scientificName: *Macroteleiabicolora* Kieffer, 1908; **Location:** country: Germany; stateProvince: Baden-Württemberg; municipality: Emmendingen; verbatimCoordinates: 48.128533°N, 7.738301°E; **Identification:** identifiedBy: Cristina Vasilita; **Event:** samplingProtocol: Malaise trap; year: 2017; month: 8; day: 2–16; **Record Level:** bibliographicCitation: *Macroteleiabicolora* (SMNS_HYM_Sce_000729, 000731); institutionCode: SMNS; basisOfRecord: PreservedSpecimen

#### Distribution

**Figure 4.**
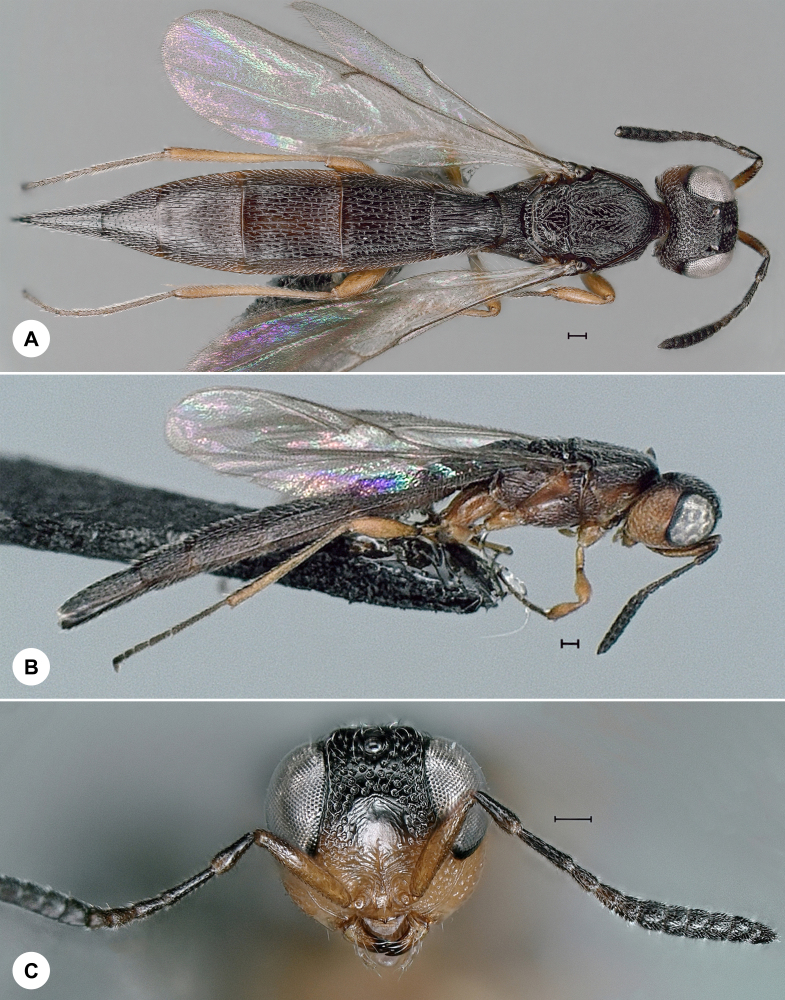
*Macroteleiabicolora* Kieffer, female, SMNS_Hym_Sce_000731. **A.** Dorsal habitus; **B.** Lateral habitus; **C.** Head, frontal view. Scale bar = 100 µm.

*Macroteleiabicolora* (Fig. 4) was described from Italy and has also been recorded from Denmark, Kazakhstan, Russia, Ukraine and the United Kingdom (Kozlov 1987, Notton et al. 2014). We here provide the first genus and species record for Germany and the first identified barcode for *Macroteleiabicolora.* Identification is based on Kozlov (1987).

### Macroteleia pannonica

Szabo, 1966

0D4C10C0-1C92-597C-9125-A5BDEB997E73

Macroteleia pannonica Szabo, 1966

#### Materials

- **Type status:** Other material. **Occurrence:** recordedBy: H.-J. Flügel; individualCount: 1; sex: female; **Taxon:** scientificName: *Macroteleiapannonica* Szabo, 1966; **Location:** country: Germany; stateProvince: Hessen; municipality: Hersfeld-Rotenburg; locality: Rockensüß, Eschkopf; verbatimElevation: 339 m; **Identification:** identifiedBy: Cristina Vasilita; **Event:** samplingProtocol: Malaise trap; year: 2012; verbatimEventDate: 25 Jul.–15 Aug. 2012; **Record Level:** bibliographicCitation: *Macroteleiapannonica* (SMNS_Hym_Sce_000159); institutionCode: SMNS; basisOfRecord: PreservedSpecimen

#### Distribution

**Figure 5.**
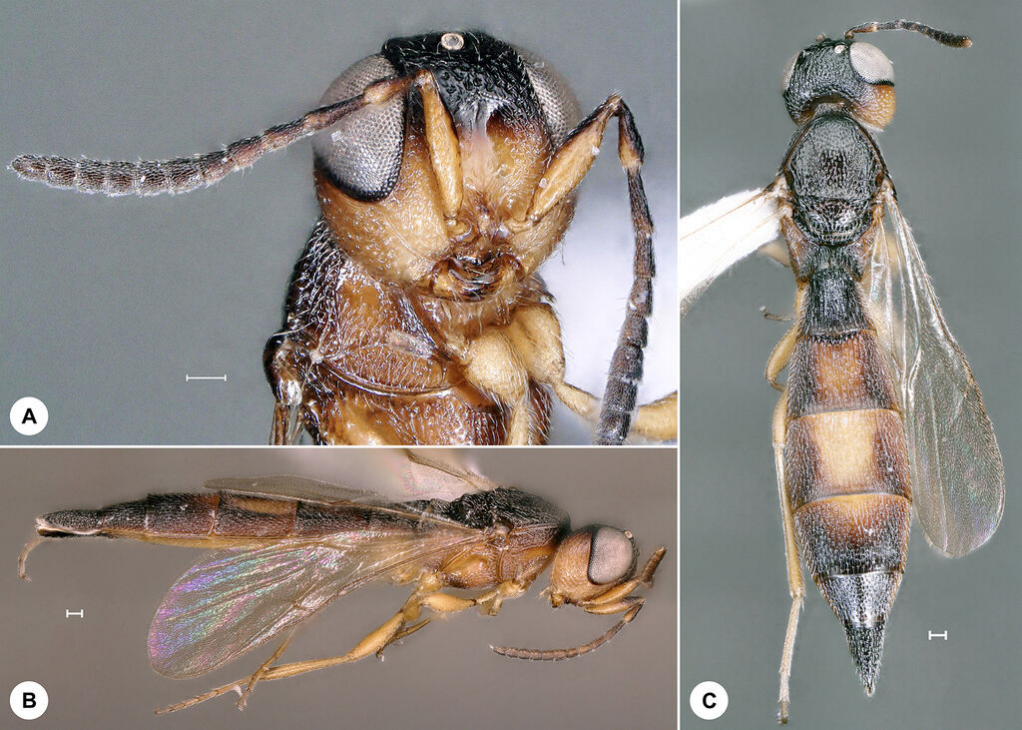
*Macroteleiapannonica* Szabo, female, SMNS_Hym_Sce_000159. **A.** Ventral head; **B.** Lateral habitus; **C.** Dorsal habitus. Scale bar = 100 µm.

*Macroteleiapannonica* (Fig. 5) was described from Hungary and has also been recorded from Romania (Fabritius and Popovici 2007, Kononova and Kozlov 2008). We here provide the first genus and species record for Germany. Identification is based on Kononova and Kozlov (2008).

### Paratelenomus saccharalis

(Dodd, 1914)

53891D70-A263-57F2-80D6-D57A6EA67B8C

Telenomus saccharalis Dodd, 1914
Liophanurus saccharalis (Dodd): Kieffer, 1926
Paratelenomus saccharalis (Dodd): Johnson, 1988

#### Materials

- **Type status:** Other material. **Occurrence:** recordedBy: L. Krogmann; individualCount: 1; sex: male; **Taxon:** scientificName: *Paratelenomussaccharalis* (Dodd, 1914); **Location:** country: Germany; stateProvince: Baden-Württemberg; municipality: Markgröningen; locality: Entomological Society of Stuttgart property in the Rotenacker; verbatimLocality: EVS-Vereinsgrundstück am Rotenacker; verbatimElevation: 280 m; **Identification:** identifiedBy: Cristina Vasilita; **Event:** samplingProtocol: sweep net; year: 2009; month: 8; day: 4; **Record Level:** bibliographicCitation: *Paratelenomussaccharalis* (SMNS_Hym_Sce_001096); institutionCode: SMNS
- **Type status:** Other material. **Occurrence:** recordedBy: T. Kothe, M. Englehardt, Ch. König; individualCount: 1; sex: female; associatedSequences: GenBank: MW829355; **Taxon:** scientificName: *Paratelenomussaccharalis* (Dodd, 1914); **Location:** country: Germany; stateProvince: Baden-Württemberg; municipality: Tübingen; locality: Wurmlingen, Gegental; verbatimElevation: 377 m; verbatimCoordinates: 48.513233°N, 8.991767°E; **Identification:** identifiedBy: Jessica Awad; **Event:** samplingProtocol: Malaise trap; year: 2014; month: 5; day: 13–23; **Record Level:** bibliographicCitation: *Paratelenomussaccharalis* (SMNS_HYM_Pla_000305); institutionCode: SMNS

#### Distribution

**Figure 6.**
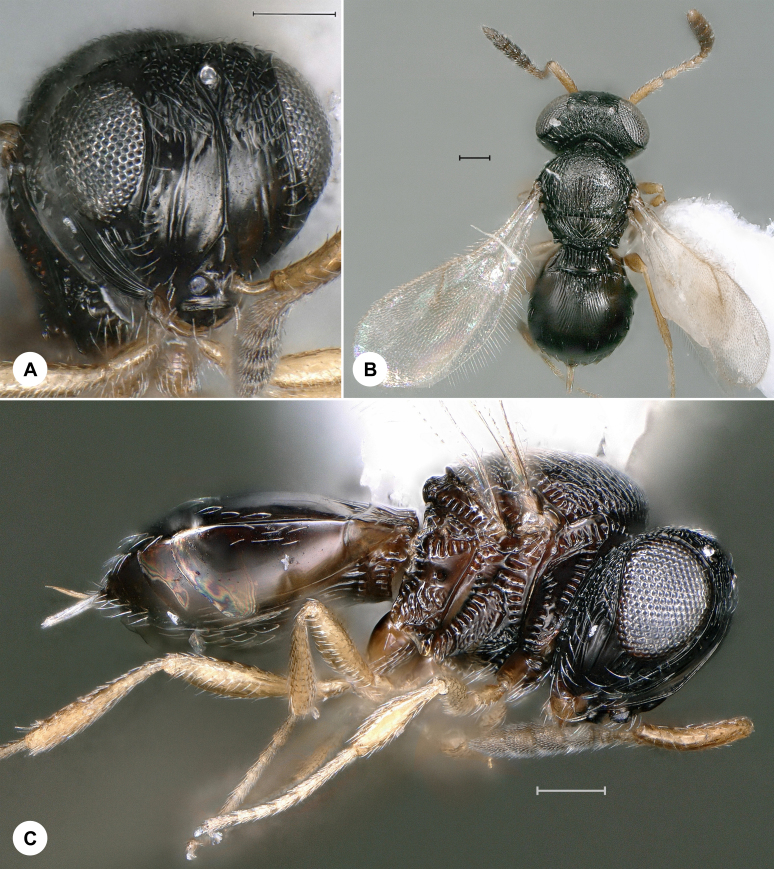
*Paratelenomussaccharalis* (Dodd), female, SMNS_Hym_000305. **A.** Head, frontal view; **B.** Dorsal habitus; **C.** Lateral habitus. Scale bar = 100 µm.

*Paratelenomussaccharalis* (Fig. 6) was described from Indonesia and has also been recorded from Australia, Austria, Bangladesh, Benin, China, Ghana, India, Ivory Coast, Italy, Japan, Kenya, Malaysia, Moldova, Nigeria, Philippines, Romania, Rwanda, Somalia, South Africa, South Korea, Taiwan, Thailand, Uganda, USA, Zambia and Zimbabwe (Johnson 1996). We here provide the first genus and species record for Germany. Identification is based on Johnson (1996).

### Probaryconus

Kieffer, 1908

E7D415C4-CDD3-55E5-ACE2-783FD3A6E875

Procacus Kieffer, 1910
Neurocacus Kieffer, 1913
Amblyconus Kieffer, 1913
Urundia Risbec, 1957
Probaryconus

#### Materials

- **Type status:** Other material. **Occurrence:** recordedBy: T. Kothe, M. Englehardt, Ch. König; individualCount: 2; sex: female; **Taxon:** scientificName: *Probaryconus* Kieffer, 1908; **Location:** country: Germany; stateProvince: Baden-Württemberg; municipality: Tübingen; locality: Wurmlingen, Gegental; verbatimElevation: 377 m; verbatimCoordinates: 48°30.794’N, 8°59.506’E; **Identification:** identifiedBy: Cristina Vasilita; **Event:** samplingProtocol: Malaise trap; year: 2014; month: 5; day: 13–23; **Record Level:** bibliographicCitation: *Probaryconus* sp. (SMNS_Hym_Sce_000344, 000345); institutionCode: SMNS

#### Distribution

**Figure 7.**
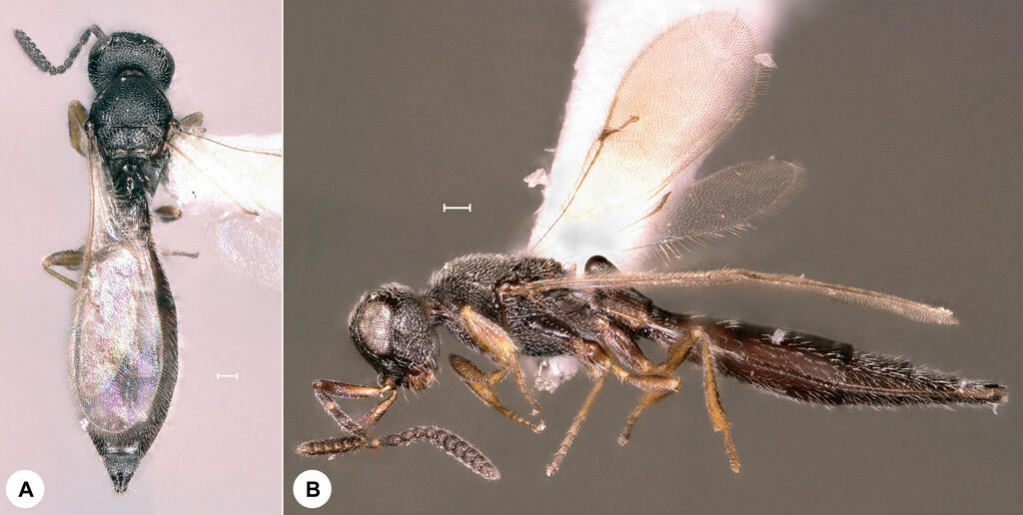
*Probaryconus* Kieffer, female, SMNS_Hym_Sce_000344. **A.** Dorsal habitus; **B.** Lateral habitus. Scale bar = 100 µm.

*Probaryconus* (Fig. 7) was described from France and has also been recorded from Australia, Azerbaijan, Belize, Benin, Botswana, Brazil, Bulgaria, Colombia, Costa Rica, Dominica, Dominican Republic, Ecuador, Egypt, France, French Guiana, Ghana, Hungary, India, Indonesia, Ivory Coast, Kenya, Kyrgyzstan, Jamaica, Madagascar, Malaysia, Mexico, Moldova, New Caledonia, Nigeria, Panama, Papua New Guinea, Paraguay, Peru, Puerto Rico, Romania, Slovakia, South Africa, Thailand, Trinidad and Tobago, Turkey, Ukraine, USA, Venezuela and the Virgin Islands (Hymenoptera Online 2020, Kieffer 1926, Kozlov 1987). We here provide the first genus record for Germany. Identification is based on Kozlov (1987) and Talamas et al. (2011).

### Trimorus varicornis

(Walker, 1836)

6E7D2762-B4C7-5892-99E9-DE48646F01BC

Teleas varicornis Walker, 1836
Teleas metabus Walker, 1836
Prosacantha minor Thomson, 1859
Prosacantha grandis Thomson, 1859
Prosacantha variicornis (Walker): Marshall, 1873
Prosacantha metabus (Walker): Marshall, 1873
Prosacantha varicornis (Walker): Walker, 1874
Prosacantha spinosa Szepligeti, 1901
Pentacantha variicornis (Walker): Kieffer, 1908
Pentacantha minor (Thomson): Kieffer, 1908
Pentacantha grandis (Thomson): Kieffer, 1908
Pentacantha rufimanus Kieffer, 1908
Pentacantha varicornis (Walker): Kieffer, 1913
Hoplogryon metabus (Walker): Kieffer, 1926
Propentacantha varicornis (Walker): Kieffer, 1926
Propentacantha minor (Thomson): Kieffer, 1926
Propentacantha grandis (Thomson): Kieffer, 1926
Propentacantha spinosa (Szepligeti): Kieffer, 1926
Propentacantha rufimanus (Kieffer): Kieffer, 1926
Trisacantha varicornis (Walker): Szabo, 1957
Trimorus grandis (Thomson): Sundholm, 1967
Trimorus minor (Thomson): Sundholm, 1967

#### Materials

- **Type status:** Other material. **Occurrence:** recordedBy: M. Hermann; individualCount: 1; sex: female; **Taxon:** scientificName: *Trimorusvaricornis* (Walker, 1836); **Location:** country: Germany; stateProvince: Baden-Württemberg; municipality: Klettgau; locality: Jestett; verbatimLocality: Flachshof BF1N; **Identification:** identifiedBy: Cristina Vasilita; **Event:** year: 1996; month: 6; day: 3; **Record Level:** bibliographicCitation: *Trimorusvaricornis* (SMNS_Hym_Sce_001100); institutionCode: SMNS

#### Distribution

**Figure 8.**
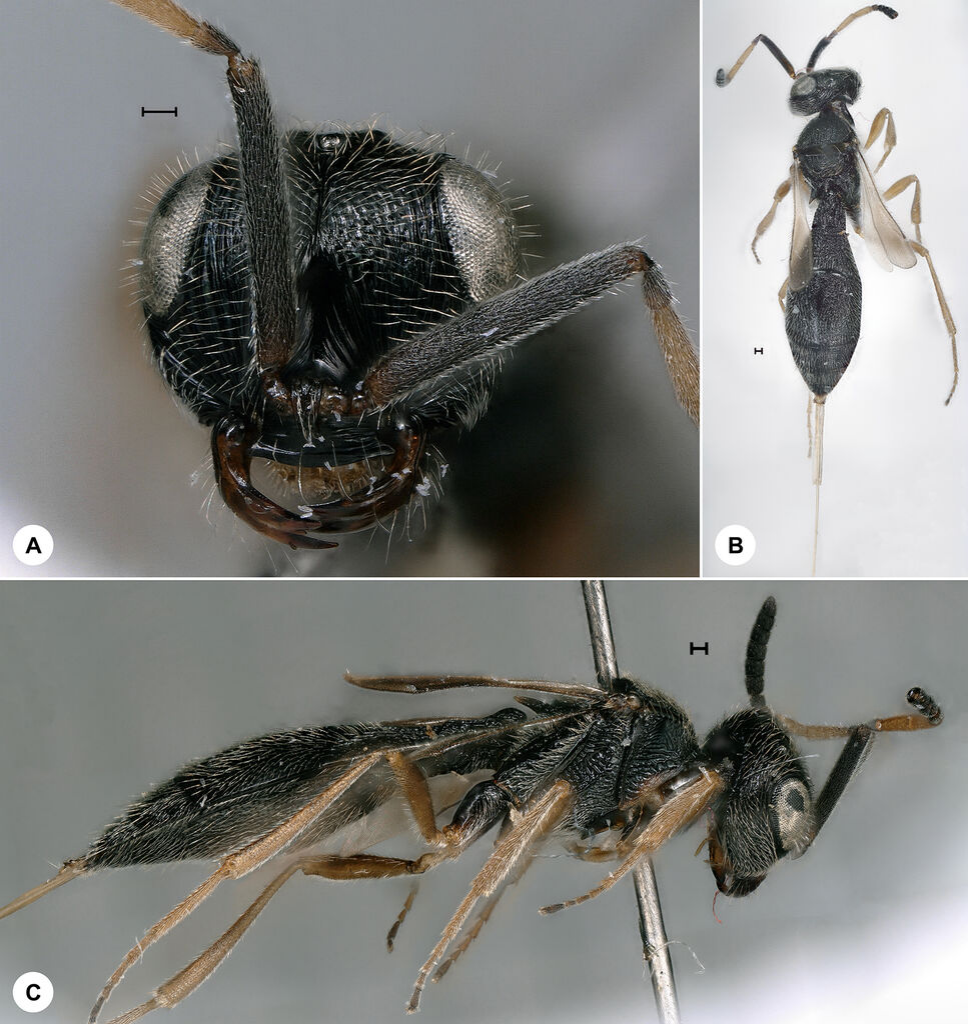
*Trimorusvaricornis* (Walker), female, SMNS_Hym_Sce_001100. **A.** Head, frontal view; **B.** Dorsal habitus; **C.** Lateral habitus. Scale bar = 100 µm.

*Trimorusvaricornis* (Fig. 8) was described from Ireland and has also been recorded from Bulgaria, Croatia, Denmark, Finland, France, Italy, Romania, Russia, Sweden, Switzerland, Ukraine and the United Kingdom (Fabritius and Popovici 2007, Hymenoptera Online 2020, Kononova and Kozlov 2001). We here provide the first species record for Germany. Identification is based on Kozlov (1987).

### Trissolcus basalis

(Wollaston, 1858)

3C3D96C9-CEE1-5B6A-8C95-B176848E3210

Telenomus basalis Wollaston, 1858
Telenomus maderensis Wollaston, 1858
Telenomus megacephalus Ashmead, 1894
Telenomus megalocephalus Schulz, 1906
Telenomus piceipes Dodd, 1920
Liophanurus megacephalus (Ashmead): Kieffer, 1926
Microphanurus africanus Fouts, 1934
Microphanurus basalis (Wollaston): Nixon, 1935
Microphanurus sulmo Nixon, 1938
Asolcus basalis (Wollaston): Delucchi, 1961
Trissolcus maderensis (Wollaston): Masner, 1965
Trissolcus piceipes (Dodd): Masner, 1965
Trissolcus sulmo (Nixon): Masner, 1965
Asolcus sulmo (Nixon): Voegele, 1969
Trissolcus africanus (Fouts): Bin, 1974
Asolcus lodosi Szabo, 1981
Trissolcus megacephalus (Ashmead): Johnson, 1983
Trissolcus lodosi (Szabo): Kononova, 2014

#### Materials

- **Type status:** Other material. **Occurrence:** recordedBy: Patricia Gut; individualCount: 2; sex: female; associatedSequences: GenBank: MW829356, MW829357; **Taxon:** scientificName: *Trissolcusbasalis* (Wollaston, 1858); **Location:** country: Germany; stateProvince: Baden-Württemberg; municipality: Freiburg; locality: Emmendingen; verbatimCoordinates: 48.128533°N, 7.738301°E; **Identification:** identifiedBy: Cristina Vasilita; **Event:** samplingProtocol: Malaise trap; year: 2017; month: 10; day: 11–25; **Record Level:** bibliographicCitation: *Trissolcusbasalis* (SMNS_Hym_Sce_000805, 000806); institutionCode: SMNS

#### Distribution

**Figure 9.**
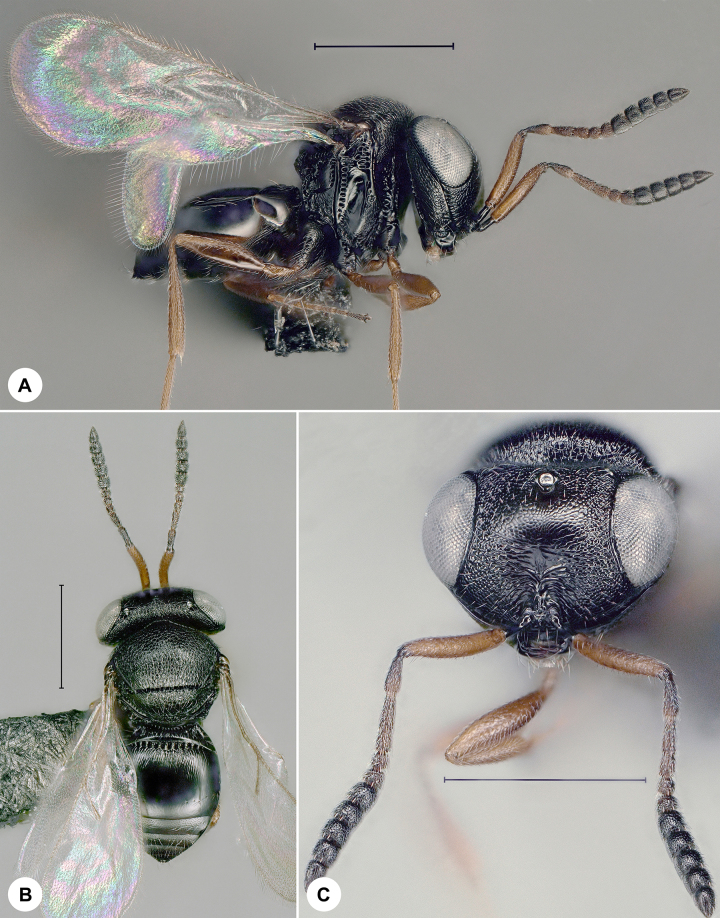
*Trissolcusbasalis* (Wollaston), female. **A.** Lateral habitus, SMNS_Hym_Sce_000806; **B.** Dorsal habitus, SMNS_Hym_Sce_000805; **C.** Head, frontal view, SMNS_Hym_Sce_000806. Scale bar = 500 µm.

*Trissolcusbasalis* (Fig. 9) was described from Portugal and has also been recorded from Australia, Brazil, China, Cyprus, France, Hungary, Iran, Israel, Italy, Jordan, Montenegro, Montserrat, South Africa, Spain, Tonga, Turkey, USA, Vanuatu and Zimbabwe Talamas et al. (2017). We here provide the first species record for Germany. Identification is based on Talamas et al. (2017).

### Trissolcus belenus

(Walker, 1836)

71A37866-C39A-51FF-877C-DE3625962366

Telenomus belenus Walker, 1836
Telenomus arminon Walker, 1836
Telenomus nigrita Thomson, 1860
Telenomus frontalis Thomson, 1860
Telenomus grandis Thomson, 1860
Telenomus nigripes Thomson, 1860
Telenomus ovulorum Thomson, 1860
Teleas pentatomae Rondani, 1877
Telenomus nigritus Thomson: Dalla Torre, 1898
Telenomus pentatomae (Rondani): Dalla Torre, 1898
Allophanurus arminon (Walker): Kieffer, 1912
Aphanurus belenus (Walker): Kieffer, 1912
Aphanurus frontalis (Thomson): Kieffer, 1912
Aphanurus grandis (Thomson): Kieffer, 1912
Aphanurus nigrita (Thomson): Kieffer, 1912
Aphanurus nigripes (Thomson): Kieffer, 1912
Liophanurus pentatomae (Rondani): Kieffer, 1912
Allophanurus arminon (Walker): Kieffer, 1926
Microphanurus belenus (Walker): Kieffer, 1926
Microphanurus frontalis (Thomson): Kieffer, 1926
Microphanurus grandis (Thomson): Kieffer, 1926
Microphanurus nigripes (Thomson): Kieffer, 1926
Microphanurus nigritus (Thomson): Kieffer, 1926
Asolcus grandis (Thomson): Masner, 1959
Trissolcus grandis (Thomson): Viktorov, 1967
Asolcus nixomartini Javahery, 1968
Asolcus silwoodensis Javahery, 1968
Trissolcus pentatomae (Rondani): Bin, 1974
Trissolcus belenus (Walker): Fergusson, 1978
Trissolcus nigripes (Thomson): Fergusson, 1978
Trissolcus nixomartini (Javahery): Fergusson, 1978
Trissolcus silwoodensis (Javahery): Fergusson, 1978
Trissolcus arminon (Walker): Fergusson, 1983
Trissolcus ovulorum (Thomson): Tortorici et al., 2019

#### Materials

- **Type status:** Other material. **Occurrence:** recordedBy: Gauss; individualCount: 12; sex: female; **Taxon:** scientificName: *Trissolcusbelenus* (Walker, 1836); **Location:** country: Germany; stateProvince: Baden-Württemberg; municipality: Hartheim Breisach; **Identification:** identifiedBy: Cristina Vasilita; **Event:** samplingProtocol: reared; year: 1971; month: 6; day: 14; habitat: ex. Heteroptera Eier [from Heteroptera eggs]; **Record Level:** institutionCode: SMNS
- **Type status:** Other material. **Occurrence:** recordedBy: T. Kothe, M. Englehardt, Ch. König; individualCount: 3; sex: female; associatedSequences: GenBank: MW829354, MW829353; **Taxon:** scientificName: *Trissolcusbelenus* (Walker, 1836); **Location:** country: Germany; stateProvince: Baden-Württemberg; municipality: Tübingen; verbatimCoordinates: 48.504317°N, 8.9956°E; **Identification:** identifiedBy: Cristina Vasilita; **Event:** samplingProtocol: Malaise trap; year: 2014; month: 7; day: 17–31; **Record Level:** bibliographicCitation: *Trissolcusbelenus* (SMNS_Hym_Sce_000713, 000716, 000719); institutionCode: SMNS

#### Distribution

**Figure 10.**
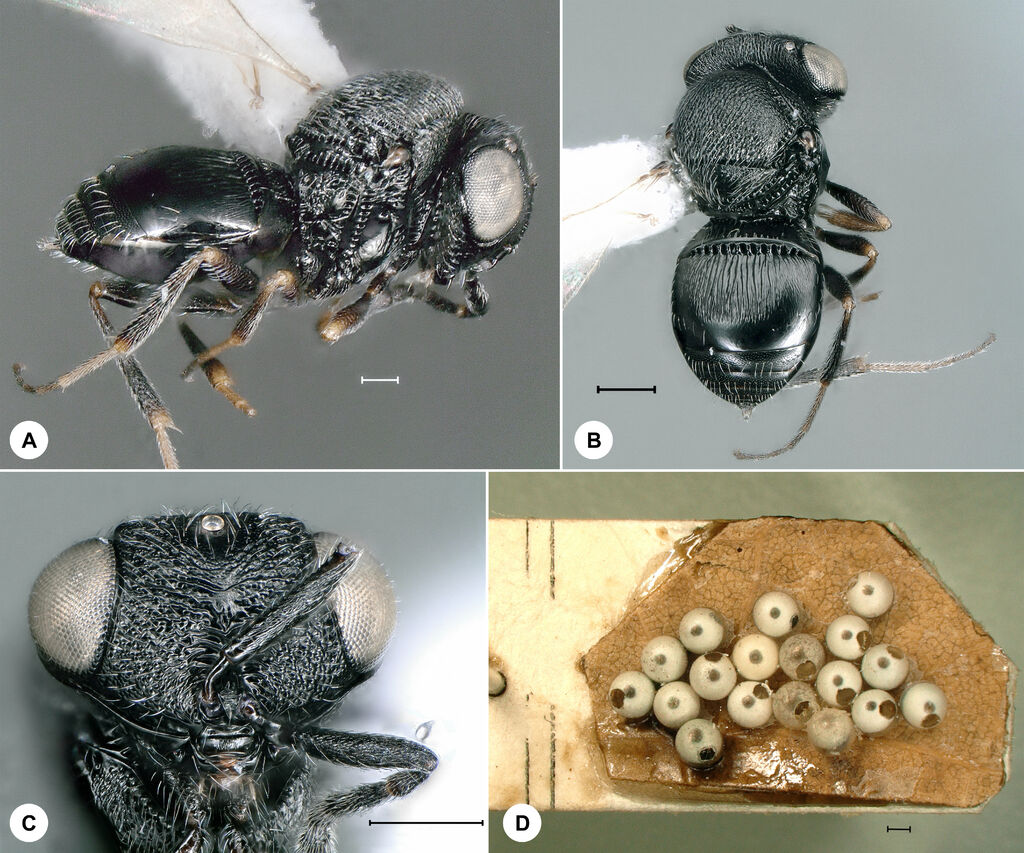
*Trissolcusbelenus* (Walker), female. **A.** Lateral habitus, SMNS_Hym_Sce_000719 (scale bar = 100 µm); **B.** Dorsal habitus, SMNS_Hym_Sce_000719 (scale bar = 200 µm); **C.** Head, frontal view, SMNS_Hym_Sce_000719 (scale bar = 200 µm); **D.** Preserved host material (scale bar = 500 µm).

*Trissolcusbelenus* (Fig. 10) was described from the UK and has also been recorded from China, France, Iran, Italy, Morocco, Portugal, Russia, Sweden, Switzerland and Tanzania (Tortorici et al. 2019). We here provide the first species record for Germany. Identification is based on Tortorici et al. 2019.

### Trissolcus colemani

(Crawford, 1912)

AE504BBC-3B9E-5B85-89F3-BF18A0193771

Telenomus colemani Crawford, 1912
Microphanurus djadetshko Ryakhovskii, 1959
Microphanurus pseudoturesis Ryakhovskii, 1959
Microphanurus rossicus Ryakhovskii, 1959
Asolcus nigribasalis Voegele, 1962
Asolcus djadetschko (Ryakhovskii): Viktorov, 1964
Asolcus pseudoturesis (Ryakhovskii): Viktorov, 1964
Asolcus bennisi Voegele, 1964
Trissolcus djadetschko (Ryakhovskii): Viktorov, 1967
Trissolcus pseudoturesis (Ryakhovskii): Viktorov, 1967
Trissolcus waloffae Javahery, 1968
Trissolcus bennisi (Voegele): Kozlov & Le, 1977
Trissolcus nigribasalis (Voegele): Kozlov & Le, 1977
Trissolcus crypticus Clarke, 1993

#### Materials

- **Type status:** Other material. **Occurrence:** recordedBy: Fischer; individualCount: 7; sex: 1 male, 6 females; **Taxon:** scientificName: *Trissolcuscolemani* (Crawford, 1912); **Location:** country: Germany; stateProvince: Baden-Württemberg; locality: Bopserwald; **Identification:** identifiedBy: Cristina Vasilita; **Event:** samplingProtocol: reared; year: 1932; month: 7; day: 12; habitat: aus Wanzeneiern [from bug eggs]; **Record Level:** institutionCode: SMNS
- **Type status:** Other material. **Occurrence:** recordedBy: Patricia Gut; individualCount: 2; sex: female; associatedSequences: GenBank: MW829352, MW829351; **Taxon:** scientificName: *Trissolcuscolemani* (Crawford, 1912); **Location:** country: Germany; stateProvince: Baden-Württemberg; municipality: Bahlingen; verbatimCoordinates: 48.128533°N, 7.738301°E; **Identification:** identifiedBy: Cristina Vasilita; **Event:** samplingProtocol: Malaise trap; year: 2017; month: 9; day: 13–27; **Record Level:** bibliographicCitation: *Trissolcuscolemani* (SMNS_Hym_Sce_000796, 000797); institutionCode: SMNS
- **Type status:** Other material. **Occurrence:** recordedBy: University of Hohenheim insect summer course; individualCount: 1; sex: male; **Taxon:** scientificName: *Trissolcuscolemani* (Crawford, 1912); **Location:** country: Germany; stateProvince: Baden-Württemberg; municipality: Tübingen; locality: Steinenberg; verbatimElevation: 460–490 m; **Identification:** identifiedBy: Cristina Vasilita; **Event:** year: 2019; month: 7; day: 1–2; **Record Level:** bibliographicCitation: *Trissolcusbelenus* (SMNS_Hym_Sce_001097); institutionCode: SMNS

#### Distribution

**Figure 11.**
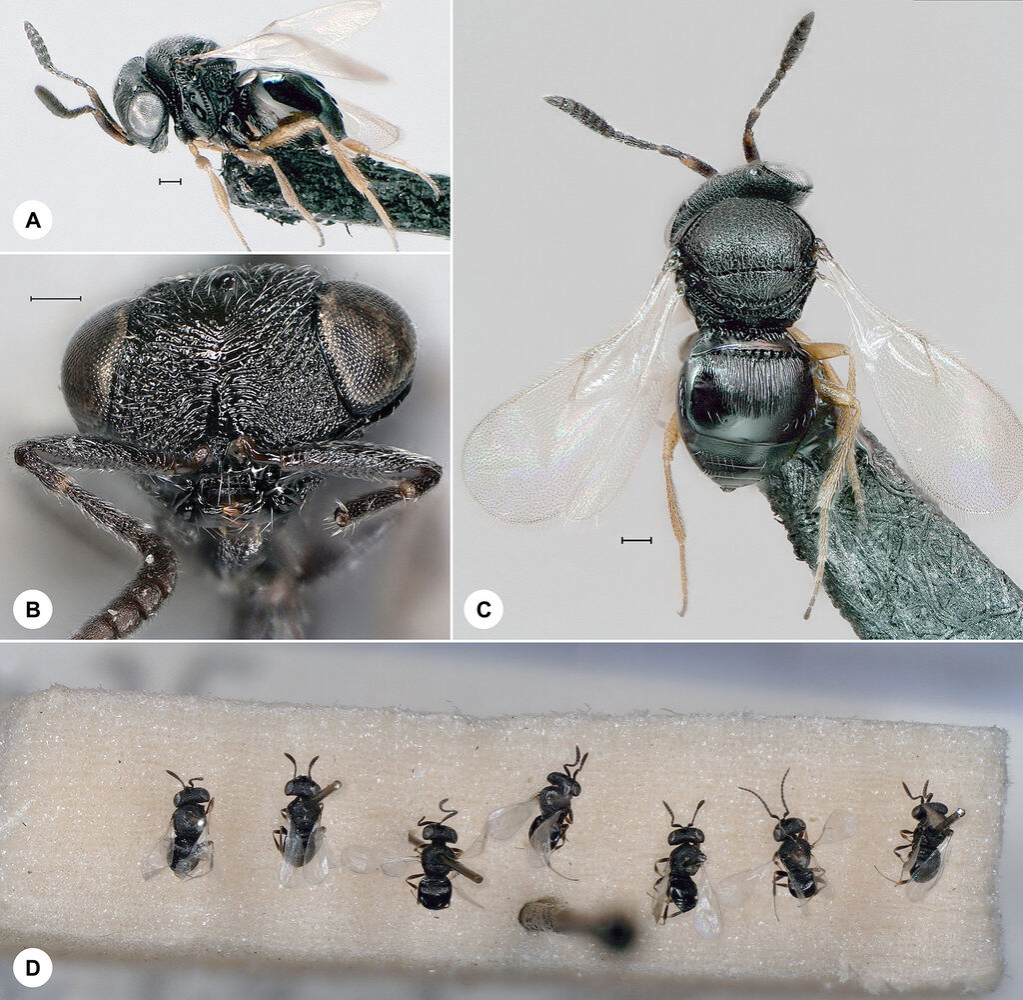
*Trissolcuscolemani* (Crawford), female. **A.** Lateral habitus, SMNS_Hym_Sce_000796; **B.** Head, frontal view; **C.** Dorsal habitus, SMNS_Hym_Sce_000797; **D.** Historical mounting method. Scale bar = 100 µm.

*Trissolcuscolemani* (Fig. 11) was described from India and has also been recorded from China, France, Greece, India, Iran, Italy, Morocco, Pakistan, Russia, Sweden, Ukraine and the United Kindgom (Tortorici et al. 2019). We here provide the first species record for Germany. Identification is based on Tortorici et al. (2019).

### Trissolcus flavipes

(Thompson, 1860)

48519DEC-BCFB-5DCF-9BE4-022E9D76D818

Telenomus flavipes Thomson, 1860
Aphanurus flavipes (Thomson): Kieffer, 1912
Microphanurus flavipes (Thomson): Kieffer, 1926
Trissolcus circus Kozlov & Le, 1976
Trissolcus crassus Kononova, 2014

#### Materials

- **Type status:** Other material. **Occurrence:** recordedBy: H.-J. Flügel; individualCount: 2; sex: female; **Taxon:** scientificName: *Trissolcusflavipes* (Thompson, 1860); **Location:** country: Germany; stateProvince: Hessen; municipality: Vogelsbergkreis; locality: Ober-Moos; verbatimLocality: Windwurffläche, SNR 5121a; verbatimElevation: 473 m; **Identification:** identifiedBy: Cristina Vasilita; **Event:** samplingProtocol: Malaise trap; year: 2012; verbatimEventDate: 29 May–18 Jun. 2012; **Record Level:** bibliographicCitation: *Trissolcusflavipes* (SMNS_Hym_Sce_000188, 000190); institutionCode: SMNS
- **Type status:** Other material. **Occurrence:** recordedBy: F. Koch; individualCount: 1; sex: female; **Taxon:** scientificName: *Trissolcusflavipes* (Thompson, 1860); **Location:** country: Germany; stateProvince: Mecklenburg-Vorpommern; municipality: Insel Rügen; locality: Kniepow; verbatimElevation: 50 m; **Identification:** identifiedBy: Cristina Vasilita; **Event:** samplingProtocol: Malaise trap; year: 2014; month: 8; day: 3–9; **Record Level:** bibliographicCitation: *Trissolcusflavipes* (SMNS_Hym_Sce_000236); institutionCode: SMNS

#### Distribution

**Figure 12.**
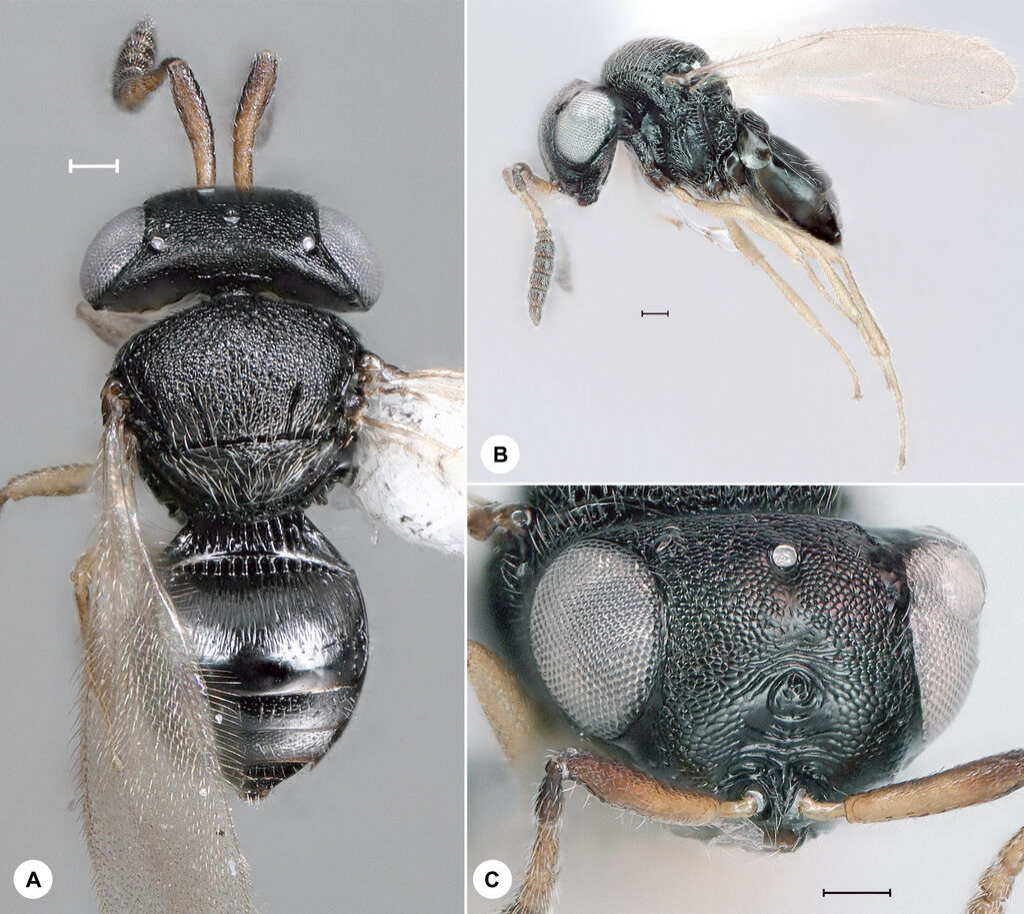
*Trissolcusflavipes* (Thompson), female, SMNS_Hym_Sce_000188. **A.** Dorsal habitus; **B.** Lateral habitus; **C.** Head, frontal view. Scale bar = 100 µm.

*Trissolcusflavipes* (Fig. 12) was described from Sweden and has also been recorded from Austria, Denmark, France, Hungary, Japan, Moldova, Romania, Russia, Sweden, Thailand, Ukraine and the United Kingdom (Talamas et al. 2017). We here provide the first species record for Germany. Identification is based on Talamas et al. (2017).

## Discussion

Of the two families of Platygastroidea, Scelionidae is better resolved. High-quality revisions and keys are available for many genera of Scelionidae, due to careful attention from professional taxonomists, as well as data regarding ecological and biological aspects. Platygastridae has been somewhat more neglected and, in large genera, such as *Platygaster* Latreille and *Synopeas* Förster, better diagnostic tools are needed for accurate species identification. This is the case with some genera of Scelionidae as well, such as *Gryon* Haliday and *Telenomus* Haliday. For example, one-hundred-year-old specimens of *Telenomus* still remain unidentified in the collection of SMNS. As taxonomic issues are resolved, it will become possible to accurately identify material for barcode reference libraries.

*Baryconuseuropaeus* and *Macroteleiabicolora* are here barcoded for the first time. A comparison with existing records in BOLD Systems (https://www.boldsystems.org/) showed no matches to identified material. For the *M.bicolora* sequences, the highest match (93.62%) was to unidentified specimens from Gabon. The *B.europaeus* sequence was most similar (97.63%) to unidentified specimens from South Africa. As expected, all *Trissolcus* sequences matched well (at least 99%) with appropriately identified material.

Based on preliminary data, several species of *Probaryconus* are found in Germany, but their nomenclature is uncertain, due to the aforementioned taxonomic impediment. Historical *Trissolcus* specimens remained unidentified in the SMNS collection for 50 to almost 100 years. The oldest of these, *T.colemani*, was reared from hemipteran eggs in 1932 (Fig. 11). A series of *T.belenus* from 1971 are preserved along with host material (Fig. 10). It is no surprise that these specimens were never identified, since *T.belenus* was largely overlooked for nearly two centuries before it was properly examined and keyed by Tortorici et al. (2019).

In addition to the newly-recorded species, *Trissolcus* species already known from Germany, such as *T.cultratus* (Mayr), *T.semistriatus* (Nees von Esenbeck) and *T.scutellaris* (Thomson), have been repeatedly detected at various locations in Baden-Württemberg. The last checklist of German Scelionidae (Dathe et al. 2001) also includes *T.choaspes* (Nixon), *T.discolor* (Ratzeburg) and *T.rufiventris* (Mayr). *Trissolcuschoaspes* is now a junior synonym of *T.scutellaris* (Thomson) (Talamas et al. 2017). The taxonomic status of *Trissolcusdiscolor* is unverifiable, as there is no known type material and some authors even debate whether *T.discolor* should be placed in *Telenomus* rather than *Trissolcus* (Kononova 2014). As for *T.rufiventris*, it was not found, which we think is an intriguing matter considering the number of *Trissolcus* specimens examined by C.V. at SMNS.

Our results emphasise that much remains to be discovered regarding parasitoid ecosystem services in Germany. Many of the newly-recorded species parasitise the eggs of stink bugs which pose a threat to vegetable and fruit production. As wasp species differ in their host preference and biological control efficacy, accurate identification is an important factor in agroecological studies (Scaccini et al. 2020). The effect of the scelionid species assemblage on local pest populations merits further attention, especially in the context of organic or sustainable food systems.

## Supplementary Material

XML Treatment for Baryconus europaeus

XML Treatment for Idris nigroclavatus

XML Treatment for Idris semiflavus

XML Treatment for Macroteleia bicolora

XML Treatment for Macroteleia pannonica

XML Treatment for Paratelenomus saccharalis

XML Treatment for Probaryconus

XML Treatment for Trimorus varicornis

XML Treatment for Trissolcus basalis

XML Treatment for Trissolcus belenus

XML Treatment for Trissolcus colemani

XML Treatment for Trissolcus flavipes
